# EZH2 in hepatocellular carcinoma: progression, immunity, and potential targeting therapies

**DOI:** 10.1186/s40164-023-00405-2

**Published:** 2023-06-02

**Authors:** Bohan Wang, Yachong Liu, Zhibin Liao, Haofeng Wu, Bixiang Zhang, Lei Zhang

**Affiliations:** 1 Hepatic Surgery Center, Institute of Hubei Key Laboratory of Hepato-Pancreato-Biliary Diseases, Tongji Hospital, Tongji Medical College, Huazhong University of Science and Technology, Wuhan, 430030 China; 2 Department of Hepatobiliary Surgery, Shanxi Tongji Hospital, Tongji Medical College, Shanxi Bethune Hospital, Shanxi Academy of Medical Sciences, Shanxi Medical University, Huazhong University of Science and Technology, Taiyuan, 030032 China; 3 Key Laboratory of Hepatobiliary and Pancreatic Diseases of Shanxi Province (Preparatory), Shanxi Tongji Hospital, Tongji Medical College, Shanxi Bethune Hospital, Shanxi Academy of Medical Sciences, Shanxi Medical University, Huazhong University of Science and Technology, Taiyuan, 030032 China

**Keywords:** HCC, EZH2, Immunity, EZH2 inhibitor

## Abstract

Hepatocellular carcinoma (HCC) is the leading cause of cancer-related death. The accumulation of genetic and epigenetic changes is closely related to the occurrence and development of HCC. Enhancer of zeste homolog 2 (EZH2, a histone methyltransferase) is suggested to be one of the principal factors that mediates oncogenesis by acting as a driver of epigenetic alternation. Recent studies show that EZH2 is widely involved in proliferation and metastasis of HCC cells. In this review, the functions of EZH2 in HCC progression, the role of EZH2 in tumor immunity and the application of EZH2-related inhibitors in HCC therapy are summarized.

## Introduction

Primary liver cancer is the sixth most common cancer and the third leading cause of cancer-related death. It ranks fifth highest in terms of its global incidence, among which 75–85% of the cases are hepatocellular carcinoma (HCC) [1]. The occurrence and development of liver cancer are complex: evidence shows that the development of liver cancer is associated with the accumulation of genetic and epigenetic changes, which mainly include DNA methylation, histone modification, and regulation of non-coding RNAs [2, 3]. Among histone modification patterns, polycomb repressive complex 2 (PRC2) is a multi-subunit protein complex. As its major catalytic subunit, EZH2 exerts epigenetic repression by catalyzing methylation of lysine 27 of histone H3 [4], resulting in inactivation of some tumor-suppressor genes or activation of oncogenes. This promotes the occurrence and progression of tumors [5, 6].

At present, surgery is the main method of treatment for liver cancer. Sorafenib and other drugs are mostly used for palliative treatment [7, 8]. Recently, the application of EZH2 inhibitors in lymphoma and breast cancer has yielded promising results in treating cancer [9, 10]. As an emerging therapeutic target, EZH2 inhibitors may be a promising combination therapy for HCC. This review summarizes the function of EZH2, the mechanism of EZH2 in liver cancer, and the current possibility of using EZH2 as a potential therapeutic target for liver cancer.

## Liver cancer

### Epidemiology

Although the etiology and pathogenesis of primary liver cancer have not been fully clarified to date, many risk factors for liver cancer have been identified, most of which can be detected and prevented at an early stage. The risk factors identified thus far mainly include genetic factors (hereditary diseases such as hemochromatosis, α1-antitrypsin deficiency, acute intermittent porphyria, porphyria cutanea tarda, and family history of HCC)[11], demographic factors (approximately 2–3 times greater incidence in males compared to females), lifestyle habits (obesity, smoking, alcohol consumption, and long-term exposure to aflatoxins), liver parenchymal lesions (cirrhosis, hepatitis B virus (HBV) or hepatitis C virus infection (HCV) infection), and metabolic diseases (non-alcoholic fatty liver, diabetes) [12]. Asia is the region with the highest liver cancer burden in the world, accounting for 72.5% of all cases. In China, the third most common cancer in men is liver cancer. The incidence and mortality rate of liver cancer have been relatively stable in recent years, but it remains one of the deadliest cancers in the world [13].

### Diagnosis and treatment

As liver cancer is a cancer with high lethality and low long-term survival rate, its early detection and treatment are crucial for the prognosis and survival of patients. Early screening for liver cancer can use a pyramid model to stratify the risk. The primary screening approach is to determine medium and high-risk groups. The commonly used methods are serum marker AFP/DCP examination, abdominal ultrasound and risk assessment models such as aMAP and cfDNA whole-genome sequencing. For fine screening of high-risk groups in primary screening, magnetic resonance imaging and cfDNA whole-genome sequencing are often adopted to identify very high-risk groups and improve early detection rates [14].

Conventional treatments for liver cancer include resection, liver transplantation, transcatheter arterial chemoembolization, radiofrequency ablation, and percutaneous absolute ethanol injection [15]. There are also some combination therapies, such as radiofrequency ablation combined with transarterial chemoembolization, combination between cytotoxic T-lymphocyte-associated protein 4 inhibitors, and PD-1/PD-L1 inhibitors [8, 16]. Moreover, nanomedicine has been found to be a promising approach in reducing the adverse effects of chemotherapy drugs. Chimeric antigen receptor T-cell therapy also offers a promising option for personalized treatment [17, 18]. Successful treatment of complex cases requires a reasonable combination of various treatment methods, and the correct treatment sequence can improve survival time and quality of life after treatment [19].

## EZH2

### Structure

**Fig. 1:**
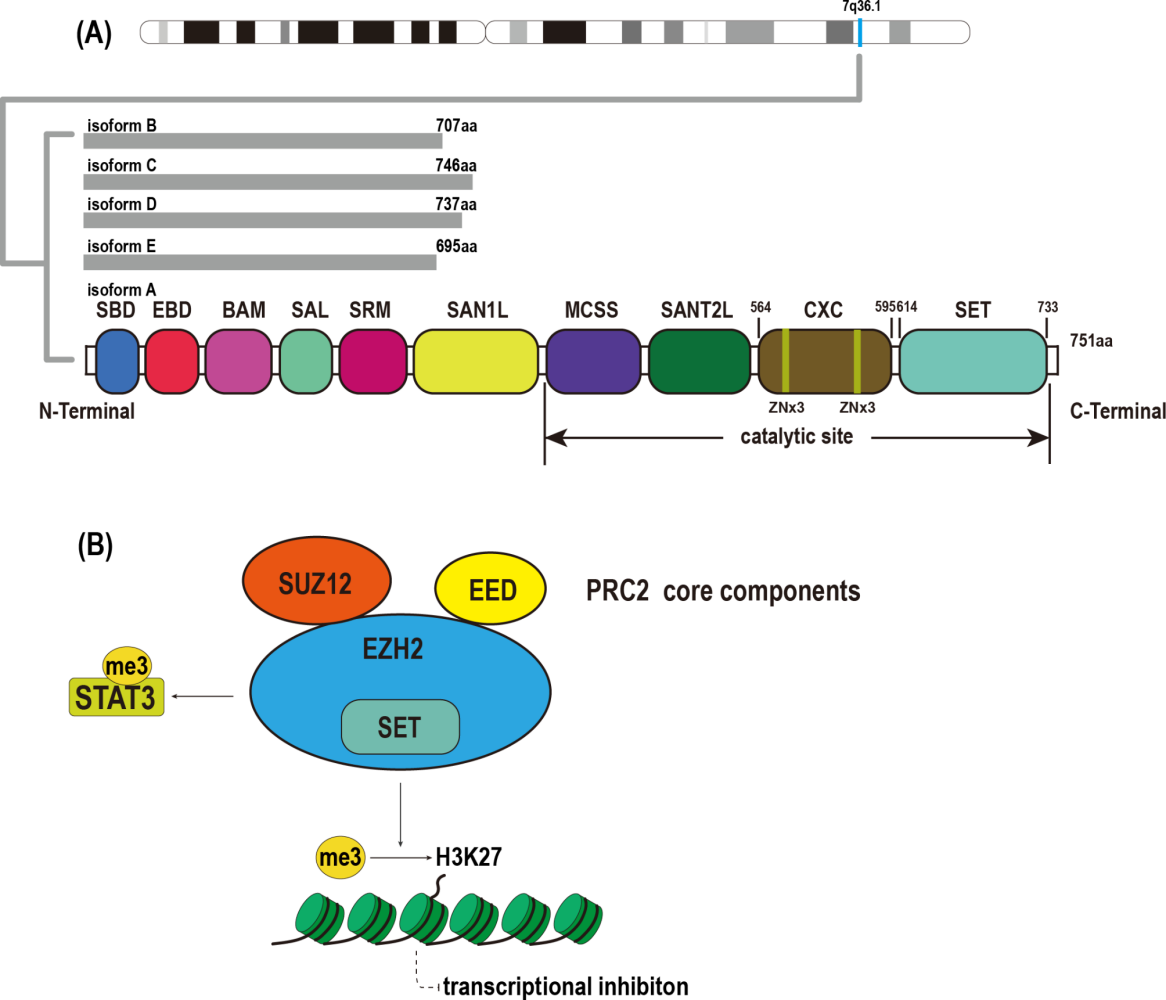
The structure and function of EZH2. **A.** The EZH2 gene is situated at the chromosomal locus 7q36.1 and has been found to encode five distinct isoforms of the protein. The amino acid lengths of five isoforms are illustrated in the figure. Schematic domain structures of EZH2 isoform A are shown, including SBD, EBD, BAM, SAL, SRM, SANT1, MCSS, SANT2, CXC, and SET. **B.** EZH2 mediates transcriptional silencing and regulates protein activity by methylating both histone and non-histone substrates

EZH2 is a core subunit of the multi-subunit protein complex PRC2, which catalyzes methylation of H3K27. PRC2 has two types of variants: PRC2.1 and PRC2.2. The core subunits of these two complexes are the same, with only auxiliary components differing [20]. EZH2 gene encodes at least five isoforms in human, of which isoform A (a 751 amino acid, 86 kDa protein) is the longest and most common transcript thereof [21, 22] (Fig. 1A).

EZH2 has 10 functional domains, which can be divided into regulatory domains near the N-terminal and catalytic domains near the C-terminal according to their function and location. Regulatory domain includes SBD, EBD, BAM, SAL, SRM and SANT1 [23]. EBD and SANT2 are the site where EZH2 can connect with the other core subunits of the PRC2 complex, EED, and SUZ12 [24] (Fig. 1B). With the stimulation of the EED and SUZ12 subunits, EZH2 can exert its methyltransferase activity. SRM is adjacent to SET in terms of the spatial conformation. By peptide binding to EED, SRM stabilizes the conformation of SET domain and transmits activation signals to SET, enabling it to act as methyltransferase [24, 25]. MCSS, SANT2, CXC, and SET make up the catalytic domain. The CXC domain interacting with DNA and nucleosome has a Zn_3_Cys_8_His cluster and a Zn_3_Cys_9_ cluster [9, 26]. SET is the main site where EZH2 exerts methyltransferase activity, and also the site of SAM (S-adenosyl-L-methionine) binding [25].

### Function

As a catalytic subunit of the PRC2 complex, one of the most common functions of EZH2 is catalyzing the trimethylation of lysine 27 of histone H3 (H3K27). This epigenetic modification of H3 usually suppresses the expression of its target genes (usually tumor-suppressor genes) [5]. For example, EZH2 represses expression of miR-22, miR-26a, and miR-622 by up-regulating the level of promoter region H3K27me3 [27–29]. In addition to histones, EZH2 can methylate some non-histone substrates and even regulate gene expression in a manner that is independent of the function of PRC2 or its methyltransferases (Fig. 1B) [5].

The non-histone substrates that EZH2 methylates are not only transcription factors but also EZH2 itself and the cofactor JARID2 of PRC2.2. Automethylation of EZH2 occurs at K510, K514, and K515 at its conserved flexible loop, which promotes the binding of EZH2 to histone H3 and enhances its activity as a histone methyltransferase [30]. EZH2-mediated trimethylation appears at the K116 site of JARID2. Methylated JARID2 regulates H3K27me3 deposition and PRC2 activity [30]. Besides, transcription factors that serve as substrates of EZH2 mainly include globin transcription Factor 4 (GATA4), STAT3, androgen receptor (AR), promyelocytic leukemia zinc finger protein, and β-catenin. Some of these factors, such as STAT3, β-catenin, retinoic acid-related orphan nuclear receptor alpha, and AR, also play an important role in the occurrence and development of HCC [5].

EZH2 can further regulate the development of immune cells, such as early development and differentiation of T and B cells, and plays an important role in the proliferation of lymphocytes, the migration of myeloid lineage cells, and other functions [31]. Given the function of EZH2 and its relationship with the immune system, EZH2 is widely reported to be involved in the tumor immune microenvironment. In general, the important role of EZH2 in cancer is the focus of much research.

## EZH2 and HCC

**Fig. 2:**
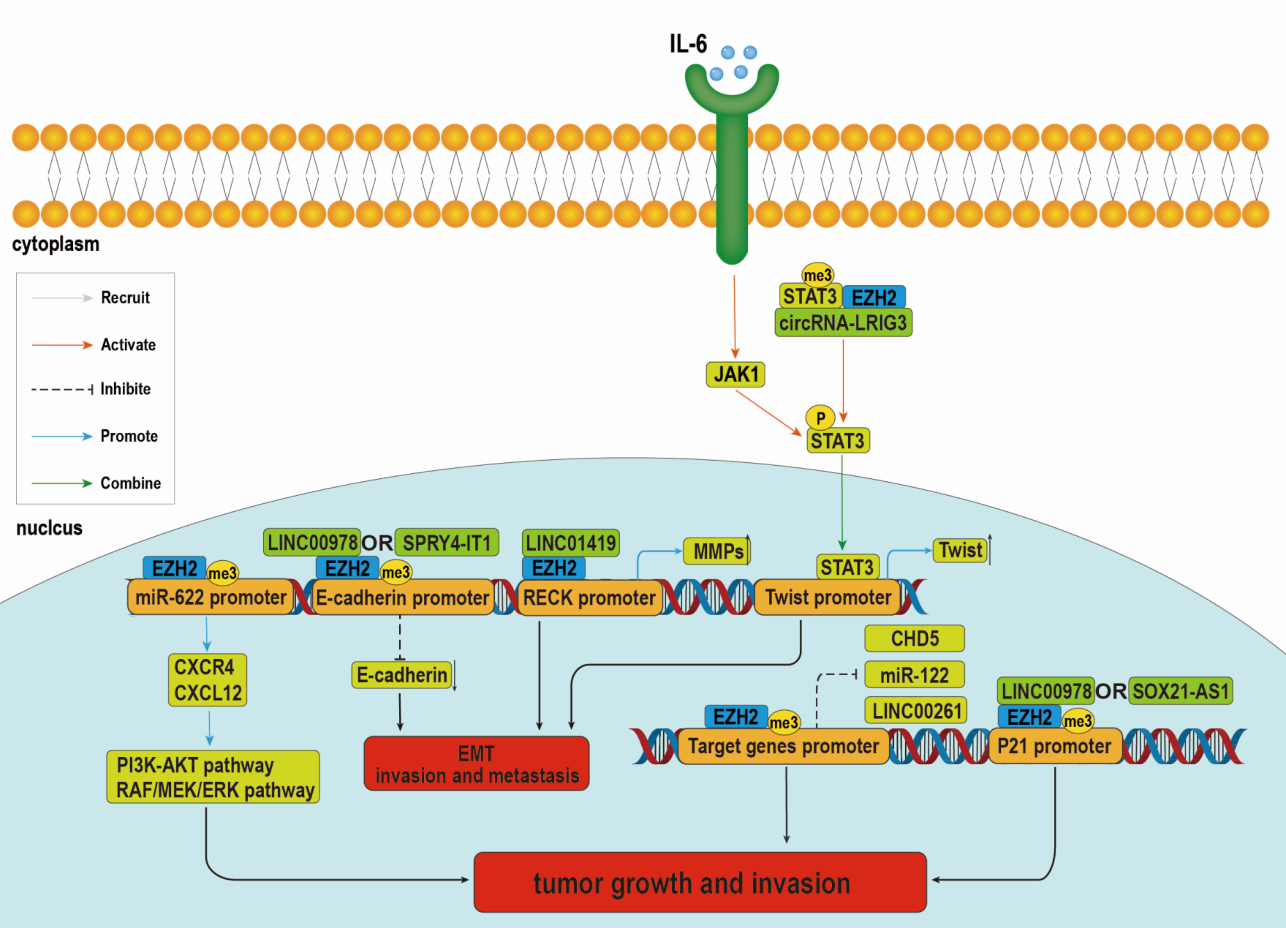
Various mechanisms by which EZH2 regulates the proliferation and metastasis of HCC cells. (i) EZH2 exerts its oncogenic effects on liver cancer through a complex interplay of molecular mechanisms. EZH2 impedes the expression of LINC00261, miR-122, LINC00978, SOX21-AS1, CHD5, and RECK via mediation of H3K27 trimethylation within their respective promoter regions, thus leading to enhanced proliferation and invasion of liver cancer cells. (ii) EZH2 also enhances CXCR4 expression by suppressing miR-622 expression to up-regulate CXCR4/CXCL12, RAF/MEK/ERK, and PI3K/ATK pathways, promoting the proliferation and metastasis of HCC cells. EZH2 is selectively recruited by LINC01419, LINC00978, and SPRY4-IT1 to the promoters of its target genes RECK and β-cadherin, causing down-regulation of β-cadherin and up-regulation of MMPs via mediating trimethylation (iii) EZH2 facilitates the methylation of STAT3 via formation of a complex with circRNA-LRIG3 and STAT3, resulting in the phosphorylation and activation of STAT3. IL-6 can activate STAT3 via the JAK/STAT3 pathway, thereby inducing the expression of Twist. In these ways, EZH2 promotes the metastasis and invasion of liver cancer cells

As early as 2005, EZH2 was found to be associated with liver cancer [32]. Previous studies have reported that EZH2 is over-expressed in HCC tissues, closely related to the poor prognosis and promotion of the progression of HCC [33]. Many studies have found that overexpression of EZH2 promoted the tumorigenesis and progression of HCC. For example, EZH2 enhanced the proliferation and metastasis of HCC cells through epigenetic silencing of multiple tumor-suppressor genes such as P21, chromodomain helicase DNA binding protein 5 (CHD5), Cdkn2a, etc.[34–36]. Moreover, EZH2 promotes BIM1-dependent hepatocarcinogenesis by silencing mircoRNA-200c [37]. Considering the oncogenic effect of EZH2 on HCC, clarifying the molecular mechanism is important in the development of novel diagnostic and therapeutic strategies for HCC. The function and mechanism of EZH2 currently found in HCC are summarized in Table 1; Fig. 2.

**Table 1:** Summary of EZH2 function and mechanism in HCC

Partner/target	Mechanism	Effect on HCC	Ref
FBP1/PKLR	EZH2 inhibits PKLR expression to inactivate NK cells residing in the liver.	promotes the occurrence and development of liver tumors.	[65]
E2F1/DDX11	DDX11 enhances the stability of EZH2 to downregulate P21. E2F1 cooperates with EZH2 to upregulate DDX11 mRNA expression.	promotes HCC cell proliferation.	[45]
CHD5	EZH2 promotes trimethylation of H3K27 by directly binding to the promoter site of CHD5, resulting in downregulation of CHD5 expression.	promotes HCC cell proliferation and invasion.	[35]
SOX21-AS1	Recruit EZH2 to the promoter of P21 and inhibit expression of P21.	promotes HCC growth and metastasis	[55]
LINC00978	Recruits EZH2 to the promoter of p21, resulting in enrichment of H3K27me3 at the promoter and suppressing p21 and E-cadherin expression.	promotes HCC growth and metastasis.	[34]
SPRY4-IT1	Recruit EZH2 to the promoter of E-cadherin and inhibit the expression of E-cadherin through enrichment of H3K23me3 at the promoter of E-cadherin.	promotes HCC cell invasion and proliferation.	[44]
FOXA2	EZH2 represses expression of LINC00261 to promote FOXA2 transcription.	promotes HCC invasion and metastasis.	[47]
LINC01419	LINC01419 binds to EZH2, resulting in H3K27me3 enrichment at the promoter region and suppresses transcription of RECK.	promotes HCC growth and metastasis	[41]
LNC-β-CATM	LNC-β-CATM associates with EZH2 and β-catenin, leading to β-catenin methylation, thereby activating Wnt-β-catenin signaling.	correlates positively with HCC prognosis and severity.	[42]
HOTAIR	Recruits EZH2 to upregulate expression of DMMTs to inhibit the expression of miR-122 to activate cyclin G1.	promotes HCC growth.	[43]
miR-622	EZH2 inhibits miR-622 expression by promoting trimethylation of H3K27 at the miR-622 promoter region.	promotes hepatoma cell growth and migration.	[29]
circRNA-LRIG3	CircRNA-LRIG3 binds to EZH2 and STAT3 to promote EZH2-induced STAT3 activation.	promotes HCC growth and metastasis.	[50]
Galectin-9	EZH2 promotes expression of Galectin-9 by inhibiting the transcription of miR-22.	promotes CD4^+^T-cell senescence.	[59]
NF-κB	EZH2 forms a complex with NF-κB (P50 and P65) under induction of CCRK to promote transcription of IL-6.	promotes immune evasion and MDSC expansion.	[52]
ULBP1	EZH2 increases ULBP1 promoter DNA methylation through DNMT3 and downregulates NKG2D ligand expression in hepatoma cells.	inhibits NK-cell-mediated cytotoxicity.	[63]
CXCL10	EZH2 suppresses transcription of CXCL10 by upregulating the level of H3K27me3 on its promoter.	attenuates NK-cell recruitment at tumor sites.	[62]

### The role of EZH2 in HCC proliferation

EZH2 is closely related to the proliferation of liver cancer cells. EZH2 was found to regulate the proliferation of HCC cells and promote HCC progression in a variety of ways, for example, previous studies have shown that CHD5 is a tumor suppressor. It promoted apoptosis and senescence and inhibits cell proliferation [38]. It was reported that EZH2 directly bound to the promoter site of CHD5 and promoted trimethylation of H3K27, resulting in down-regulation of CHD5 and promoting tumor growth in HCC [35].

CXC chemokine receptor 4 (CXCR4) is a G protein-coupled receptor that exerts multiple functions at different stages of HCC progression. EZH2 inhibited the expression of miR-622, an upstream regulator of CXCR4, by promoting trimethylation of H3K27 at the miR-622 promoter. This increases the level of expression of CXCR4, promoting the progression of HCC. In combination with CXCL12, CXCR4 regulated the PI3K-AKT pathway and RAF/MEK/ERK pathway to promote the proliferation of HCC cells [29].

Various types of non-coding RNAs (ncRNAs) play important regulatory roles in the development of liver tumors [39, 40]. NcRNAs were reported to bind to EZH2 and recruit it to the effect of regulating downstream genes, for example, LINC01419 interacted with EZH2 to inhibit the expression of RECK. Silenced RECK was associated with a poor survival rate in HCC patients. On one hand, LINC01419 recruited RNA binding protein FUS to interact with EZH2-mRNA, reducing the rate of degradation of EZH2-mRNA. On the other, LINC01419 bound to EZH2 to form the LINC01419-EZH2 complex. This complex bound to the promoter of RECK, inhibiting the transcription of RECK through H3K27me3. The down-regulation of RECK promoted the proliferation and metastasis of hepatoma cells [41]. EZH2 bound to LNC-β-CATM and β-catenin to form a ternary complex, promoting methylation of K49 at the N-terminus of β-catenin. Ubiquitination of methylated β-catenin was inhibited, activating Wnt-β-catenin signaling and promoting hepatic stem cell self-renewal and tumor growth *in vivo* [42].

Cyclin G1 is a cell cycle protein that is negatively regulated by miR-122. LncRNA HOTAIR promoted cell proliferation and HCC development by recruiting EZH2 to up-regulate the expression of DNMTs and inhibit the expression of miR-122 through DNMTs-mediated DNA methylation, activating the oncogene cyclin G1 [43].

P21 is a cyclin-dependent kinase (CDK) inhibitor that inhibits the activity of the CDK complex and thus negatively regulates the cell cycle. It is also involved in DNA replication and DNA damage repair. Both LINC00978 and SOX21-AS1 were reported to bind to EZH2 and recruit it to the P21 promoter, leading to enrichment of H3K27me3, transcriptionally inhibiting expression of P21 and promoting tumor growth and metastasis *in vivo* [34, 44].

EZH2 has also been found to promote the proliferation of HCC cells through the E2F1/DDX11/EZH2 positive feedback loop. DDX11 is a DNA helicase that promotes cell proliferation without affecting metastasis by up-regulating the stability of EZH2 to down-regulate P21 expression. After knocking-down DDX11, the EZH2 ubiquitination level was increased and P21 expression was significantly up-regulated. The cell cycle finally arrested in the G1 phase. E2F1 that is an upstream regulator of DDX11 can directly bind to the promoter of DDX11 and up-regulate DDX11 expression in HCC cells at the transcriptional level. EZH2 can also cooperate with E2F1 to up-regulate DDX11 at the mRNA level [45].

### The role of EZH2 in the invasion and metastasis of HCC cells

The epithelial-mesenchymal transition (EMT) plays an important role in the invasion and metastasis of HCC. Due to the transformation from an epithelial to a mesenchymal phenotype, malignant hepatocytes acquire the ability to produce angiogenic factors, which can enhance resistance to apoptotic stimuli such as hypoxia. Hepatoma cells undergoing EMT have a significantly increased ability to metastasize and invade [46].

FOXA2, a protein of the fork-head box transcription factor family, has been shown to suppress metastasis in HCC. LINC00261 activated the transcription of FOXA2 gene by recruiting SMAD3 to the promoter region of the FOXA2, promoting its expression. This in turn inhibited EMT and metastasis of HCC cells. EZH2 inhibited the expression level of LINC00261 by up-regulating the H3K27me3 level in the promoter region of LINC00261 and thus promoted HCC invasion and metastasis [47].

EZH2 promotes the EMT process by up-regulating expression of Twist. Twist is an important transcription factor that regulates the EMT process [48]. STAT3 had been demonstrated to bind the Twist promoter and promote its expression [49]. EZH2 not only directly formed a ternary complex with circRNA-LRIG3 and STAT3 to activate STAT3 [50] but also formed an EZH2-NF-κB complex with NF-κB and bound to the IL-6 promoter to promote its expression. Through the IL-6/JAK/STAT3 axis, EZH2 indirectly activated STAT3 to activate the EMT pathway [51–53].

In addition, E-cadherin, a marker of epithelial cells, is a key molecule for EMT[54]; loss of the adhesion link component E-cadherin led to increased tumor invasiveness and promotes HCC metastasis [34, 55]. Both SPRY4-IT1 and LINC00978 can recruit EZH2 to the promoter of E-cadherin, inhibiting expression of E-cadherin and facilitating the invasion and metastasis of HCC cells [34, 44].

The TGF-β signaling pathway is known to play an important role in HCC initiation and EMT [56]. EZH2 promoted CXCR4/CXCL12 signaling pathway through inhibition of miR-622 expression. The cross-talk between CXCR4/CXCL12 pathway and TGF-β signaling inhibited the E-cadherin expression and activated the EMT pathway, enhancing the ability of tumor invasiveness in HCC cells [57, 58].

### The role of EZH2 in tumor immunity

**Fig. 3:**
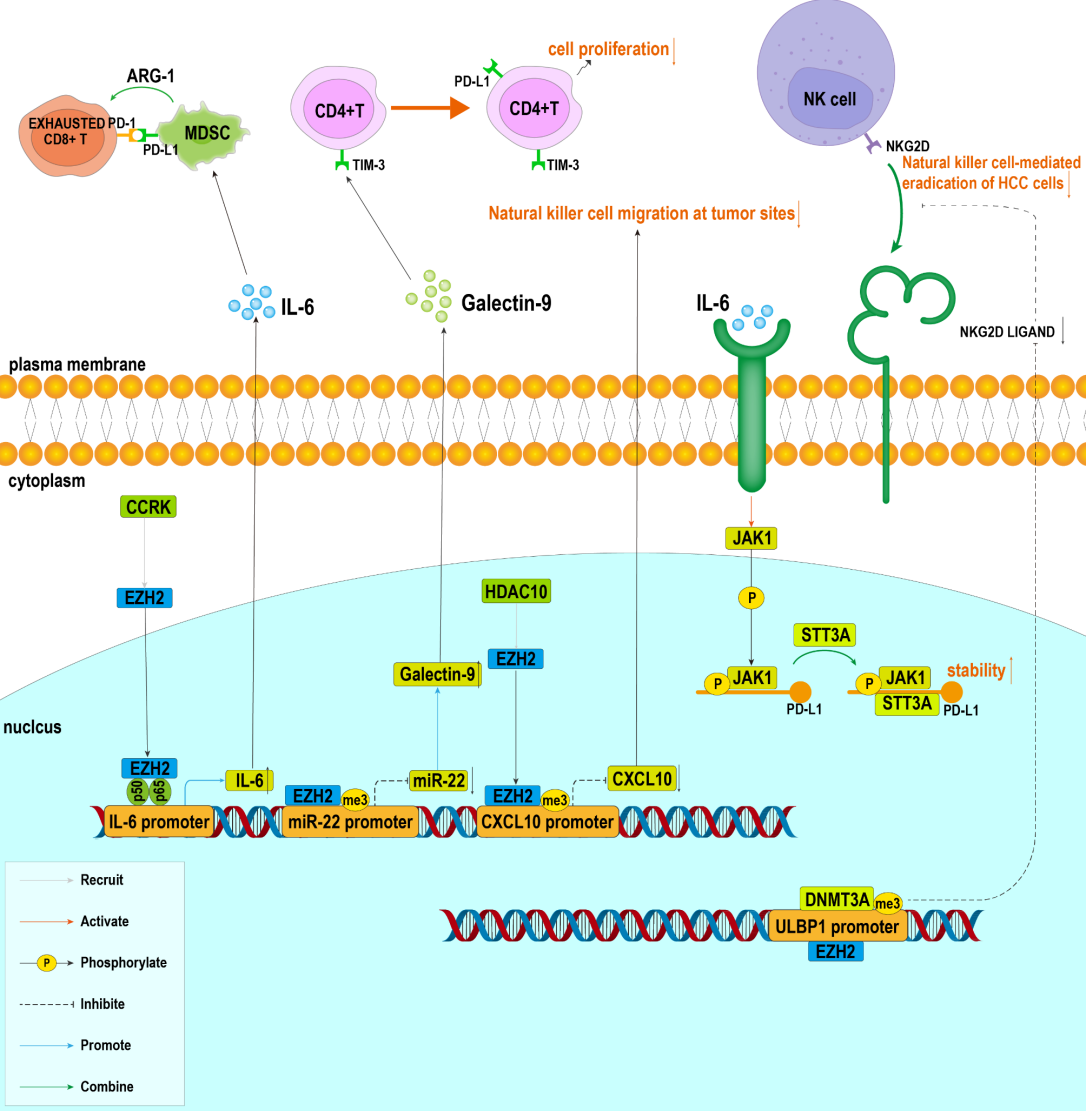
Signaling pathways associated with EZH2 in HCC immunity. Among them, EZH2 inhibits CD4^+^ T cell proliferation by inhibiting miR-22 to activate the Galectin-9/TIM-3 pathway. EZH2 also down-regulates expression of the NKG2D ligand to resist NK-cell-mediated cytotoxicity. EZH2 promotes IL-6 expression with the involvement of CCRK and NF-κB. IL-6 functions in two different ways: promoting the expansion of MDSC; activating JAK1 to increase the glycosylation levels of PD-L1, thereby up-regulating its stability

In addition to affecting tumor proliferation and metastasis, EZH2 is also involved in tumor immunity, including immune evasion, NK-cell-mediated cytotoxicity, and T-cell function. This section focuses on the function of EZH2 on tumor immunity in HCC (Fig. 3).

#### The effect of EZH2 on T cells

T cells play an important role in antitumor immunity. EZH2 exerts its cancer-promoting role by promoting T-cell senescence and immune evasion. In HBV-associated HCC, EZH2 promoted expression of Galectin-9 by inhibiting the transcription of miR-22 [28]. Galectin-9 constituted the Galectin-9/TIM-3 pathway with TIM-3 that is on the surface of CD4^+^ T cells in HBV-related HCC. The pathway promoted T-cell senescence and suppression, which led to a decreased killing capacity among T cells. Furthermore, the expression of cell cycle-dependent kinase inhibitors was increased in TIM-3^+^CD4^+^ T cells. This finding suggested that TIM-3^+^CD4^+^ T cells failed to enter the cell cycle (in any active sense) and its proliferative capacity was also limited. Li et al. used TIM-3^+^CD4^+^ T cells and Galectin-9^+^ KCs for *in vitro* co-culture. The isotype controls supplemented with anti-TIM-3 mAb increased T-cell proliferation, as well as the expression of T-cell effector molecules IL-2 and IFN-γ. Blockade of the Galectin-9/TIM-3 pathway restored effector T-cell function, which further demonstrated that EZH2 promoted immune evasion through the Galectin-9/TIM-3 pathway [59].

EZH2 inhibited T-cell killing activity and promoted immune evasion through formation of the EZH2-NF-κB (p65 and p50) complex induced by CCRK. The component promoted transcription of IL-6 by binding to the IL-6 promoter. IL-6, in turn, activated JAK1 in HCC cells, leading to phosphorylation of the Y112 residue of non-glycosylated PD-L1 in the endoplasmic reticulum. Subsequent binding between phosphorylated PD-L1 and the glycosyltransferase STT3A was enhanced, initiating the glycosylation of PD-L1 and elevating its stability. As an immune checkpoint protein, PD-L1 bound to the PD-1 receptor on T cells, inhibited cytokine secretion from T lymphocytes and promoted T lymphocyte apoptosis [52, 60]. IL-6 also promoted the expansion of myeloid-derived suppressor cells (MDSCs), which increased the expression of immunosuppressive products such as ARG-1, nitric oxide synthase, and reactive oxygen species, inhibiting the activity of immune cytotoxic T cells and depleting CD8^+^ T cells [61]. Thus, EZH2 suppresses T-cell-associated tumor immunity to promote progression of HCC.

#### The effect of EZH2 on natural killer (NK) cell-mediated tumor clearance

In NK-cell-mediated tumor-killing immunity, NK cells recognize NK-cell ligands on target cells to exert their cytotoxic effect. As an NKG2D ligand, ULBP1 played an important role in NK-cell-mediated cytotoxicity in HCC. EZH2 recruited DNMT3A to the promoter of ULBP1, increasing DNA methylation and thus down-regulating the expression of NKG2D ligands to resist NK-cell-mediated cytotoxicity in hepatoma cells. As a chemokine, CXCL10 promoted the recruitment of NK cells to tumor sites, exerting a tumor-suppressive effect through NK-cell-mediated tumor cell growth inhibition without affecting the killing activity of NK cells on hepatoma cells. EZH2 was recruited to the CXCL10 promoter by HDAC10, inhibiting CXCL10 transcription by up-regulating the level of expression of H3K27me3. Due to down-regulation of CXCL10 expression level, the migration of NK cells at tumor sites was decreased and NK-cell-mediated tumor growth inhibition was attenuated [62, 63]. Additionally, studies found that EZH2 regulated the number of NK cells in the liver by controlling the metabolic process. Fructose-1,6-bisphosphatase 1 (FBP1) is a rate-limiting enzyme in gluconeogenesis. Liao et al. found that attenuation of FBP1 was partly caused by EZH2-mediated promoter hypermethylation [64]. PKLR was a key protein transmitted to NK cells through extracellular vesicles and regulated the function of, and glycolysis in, NK cells. EZH2 inhibited the expression of PKLR when FBP1 was deleted in hepatocytes, resulting in a significant decrease in the level of PKLR in extracellular vesicles produced by hepatocytes and inactivation of NK cells residing in the liver.The tumorigenesis and progression of liver tumors were ultimately promoted [65].

## Targeting EZH2 drugs and strategy

In recent years, targeted therapies have become a popular area of research for scientists. Current findings show that EZH2 mainly functions as an oncogenic factor in the tumorigenesis and progression of HCC [66]. Therefore, the targeted inhibition of EZH2 or its upstream and downstream target genes may provide an effective therapeutic strategy for HCC.

### Inhibitors against EZH2

**Fig. 4:**
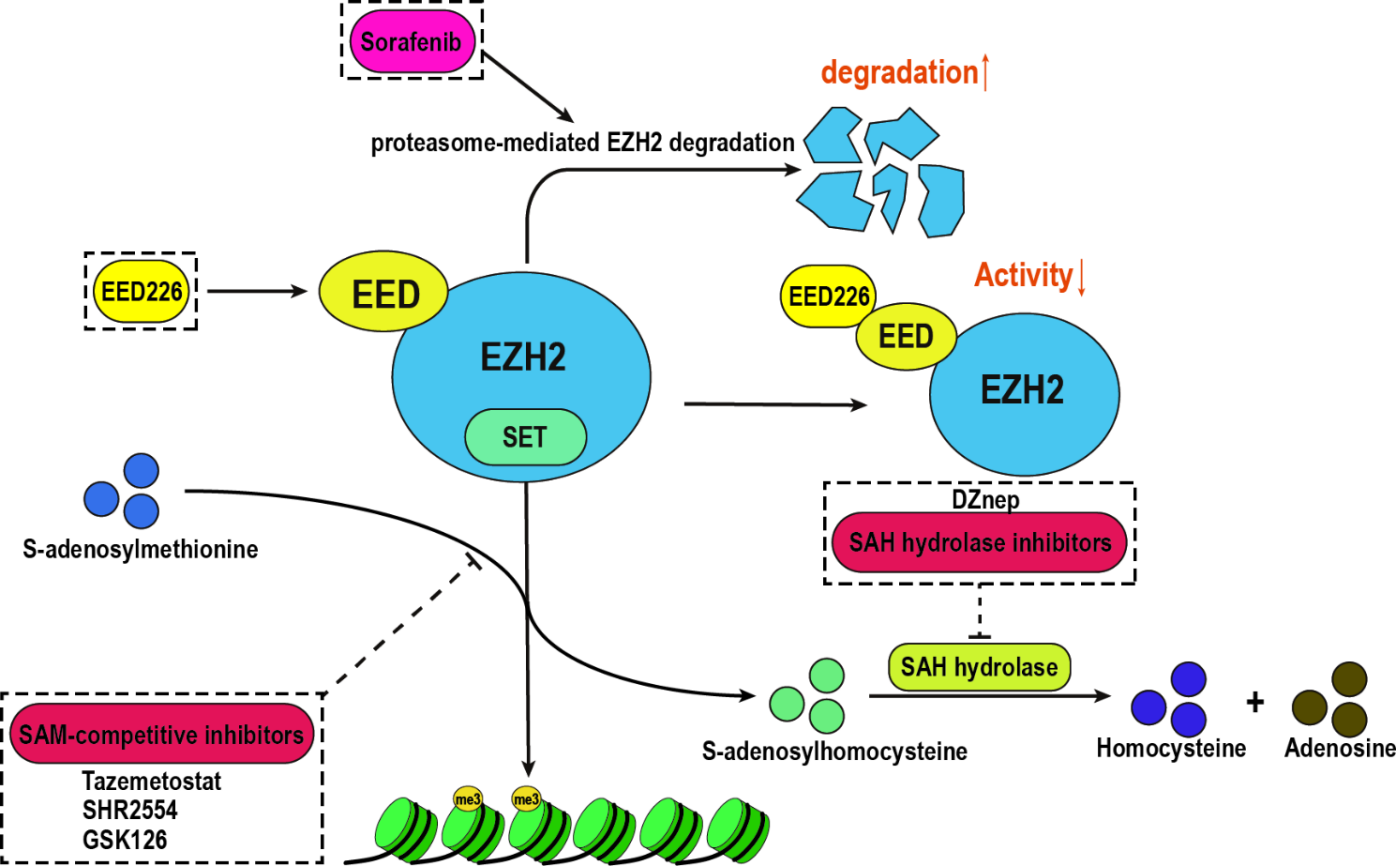
Classification and mechanism of EZH2 inhibitors. Three different types of EZH2 inhibitory mechanisms are demonstrated: inhibition of the methyltransferase activity of EZH2, promotion of EZH2 degradation, and disruption of the interaction among EZH2 and other PRC2 components

Various EZH2 inhibitors have been reported. According to different targeting mechanisms, they can be divided into three categories: inhibiting methyltransferase activity of EZH2, disrupting the interaction among EZH2 and other PRC2 components, and promoting EZH2 degradation (Table 2; Fig. 4). Commonly used inhibitors, such as 3-deazaneplanocin A (DZNep), EI1, GSK126 and tazemetostat (EPZ-6438) are SAM-competitive inhibitors. CPI-1205, CPI-0209, SHR2554, PF-06821497, and valemetostat (DS-3201b) are being investigated in cancer-related clinical trials [67]. DZnep, an S-adenosylhomocysteine hydrolase inhibitor, globally reduced the methylation modification of histones, such as H3K27me3, by inhibiting SAM-mediated methylation transfer [68]. It significantly inhibited self-renewal of tumor-initiating HCC cells and also showed good antitumor effects in xenograft models [69].

**Table 2:** Mechanism and clinical trial phase of drugs against EZH2

Mechanism of action	Name	Specific for EZH2	Status	Ref
SAH hydrolase inhibitors	DZnep	No	Pre-clinical	[92]
SAM-competitive inhibitors	Tazemetostat	Yes	Phase II	[93]
	GSK126 (GSK2816126)	Yes	Phase I	[94]
	SHR2554	Yes	Phase I	[76]
Interfering the interaction between EZH2 and EED	EED226 (MAK683)	Yes	Phase I	[77]
Promoting EZH2 degradation	Sorafenib	No	FDA approval	[79]

Some EZH2-selective inhibitors, such as tazemetostat, GSK126, and SHR2554, have also been developed. Tazemetostat is the first FDA approved oral highly selective small-molecule EZH2 inhibitor applied in advanced epithelioid sarcoma and follicular lymphoma. Tazemetostat competitively bound to the SET domain of EZH2 to inhibit the binding of SAM. Clinical research showed that tazemetostat exhibited good antitumor activity in patients with relapsed or refractory B-cell non-Hodgkin lymphoma (B-NHL) and solid tumors, INI1-negative malignant rhabdoid tumors, and ovarian rhabdoid tumors with SmarCA4 deletion [10, 70–72]. GSK126 and SHR2554 are SAM-competitive inhibitors currently under clinical trial. GSK126 was more than 1000 times more selective for EZH2 compared to other methyltransferases and significantly inhibited cell proliferation in diffuse large B-cell lymphoma cell lines harboring Y641F, Y641N, or A677G EZH2 mutations. Xenograft models also showed good antitumor effects, however, GSK126 was found to negatively affect antitumor immunity by increasing MDSCs and decreasing the number of CD4^+^ and IFNγ^+^ CD8^+^ T cells [73, 74]. SHR2554 was a highly selective EZH2 inhibitor that could be taken orally, showing good inhibitory activity against both Y641 mutant and wild-type EZH2 methyltransferase activity in diffuse large B-cell lymphoma cell lines. It also showed a high efficacy rate (69%) in EZH2-mutant lymphoma [75, 76].

In addition to inhibitors targeting the catalytic domain of EZH2, scientists have also developed inhibitors targeting the interaction between subunits of the PRC2 complex. For example, EED226 bound to the H3K27me3 binding pocket of EED to form an EED226-EED-EBD ternary complex, which changed the conformation of EED and led to the inhibition of PRC complex activity. In the subcutaneous transplantation model of B-NHL cell line karpas422, the tumor growth was also inhibited at a dose of 4 mg/kg administered by gavage; the tumor completely disappeared at a dose of 40 mg/kg administered for 32 days, suggesting good anti-tumor effect. In EZH2 Y111N and F120L mutated drug-resistant WSU-DLCL2 cells, EED226 inhibited cell proliferation and H3K27me3 level. EED226 could directly bind PRC2 complex and meanwhile it does not affect the binding of SAM competitive inhibitors with EZH2. Therefore, EED226 has a synergistic effect with SAM competitive inhibitors, which may be a promising combination therapy strategy [77].

As mentioned, research on EZH2 inhibitors has yielded some encouraging results, however, only DZnep and GSK126 have been used in HCC cell lines and xenograft models [69, 78]. The application of most selective EZH2 inhibitors in HCC is at the preliminary stage and requires further study.

### Drug resistance and combination therapies in HCC

EZH2 also plays an essential role in drug resistance in HCC. Sorafenib (a multikinase inhibitor) is a first-line drug used in treatment for advanced HCC. It accelerated proteasome-mediated EZH2 degradation in HCC cells to inhibit HCC proliferation [79]. It was disappointing that only approximately 30% of patients could benefit from sorafenib treatment. This population often developed resistance after response within six months [79, 80]. Researchers found that combination therapies might help to overcome drug resistance. EZH2 knockdown by siRNA in hepatoma cells increased the sensitivity to sorafenib and thus decreased the viability of tumor cells. Treatment with DZnep improved the sensitivity of hepatoma cells to sorafenib [79]. Studies further indicated that IGF1R promoted sorafenib resistance through the RAS/RAF/ERK signaling pathway and siIGF1R partially blocked EZH2-induced sorafenib resistance in HepG2 cells. This might be a new approach to deal with sorafenib resistance [81].

In addition, high expression of EZH2 was observed in the human liver multidrug-resistant cancer cell line Bel/FU. With EZH2 siRNA treatment, the killing effect of 5-FU was significantly improved. The proportion of cells arrested in the G1 phase was higher when combinaing both treatments compared to usingtheir individually. This may due to the decreased expression of MDR1 after EZH2 depletion, which decreased the ability of cells to excrete chemotherapeutic drugs and enhanced the killing effect of 5-FU on Bel/FU cells [82]. DNA methyltransferase inhibitors can reverse DNA hypermethylation. The study of Zhang et al., demonstrated the synergistic growth inhibitory effects of 5-Aza-CdR (DAC) and GSK126 in SNU5, HepG126, and SNU2 hepatoma cell lines. Compared with single application, the combined use of DAC and GSK126 in the three HCC cell lines resulted in down-regulation of stemness pathway genes and up-regulation of immune response genes, as well as a more pronounced activation of immune response [83].

### Potential strategies targeting EZH2 in HCC

Scientists have found that down-regulation of some miRNAs leads to up-regulation of EZH2 in cancer [84], therefore restoring miRNA expression might be an effective HCC therapy. In miR-26a-overexpression of HepG2 cells, stable overexpression of miR-26a down-regulated the level of EZH2, resulting in cell cycle arrest at the G1 phase and inhibition of HCC cell proliferation [27].

OGT, which catalyzes O-GlcNAc on EZH2 and thus reduces its stability by increasing the level of ubiquitination, was confirmed as a direct target of miR-15a. Furthermore, overexpression of miR-15a inhibited the migration and proliferation of Huh-7 and HCC-LM3 cells, inducing cell cycle G0/G1 arrest and promoting apoptosis in HCC cells. In addition, P53 promoted the expression of miR-15a, however, the mechanism of action thereof remains unclear and warrants further investigation as a potential therapeutic strategy [85].

Natural compound derivatives are also found to have potential value in application as EZH2 inhibitors in HCC. Emodin succinyl ester is a new derivative obtained by adding succinyl radicals to anthraquinone emodin, the active ingredient of the variety of rhubarb used in traditional Chinese medicine. In HCC cells, EZH2 up-regulated androgen receptor expression and increased the proliferation and migration of HCC cells. A study by Khan et al. suggested that emodin succinyl ester inhibited the proliferation and migration of HCC cells by targeting the interaction between EZH2 and androgen receptor to inhibit progression of HCC [86].

20(S)-Ginsenoside Rh2 (20(S)-GRh2) is a plant glycoside with a dammarane skeleton. Expression of EZH2 was found to be significantly downregulated in HepG2 and Hep3B cells treated with 20(S)-GRh2. Modification of H3K27me3 in the promoter region of the CDKN2A-2B gene cluster and enrichment of EZH2 were reduced, which increased mRNA levels of the tumor-suppressor genes P14, P15, and P16 and inhibited HCC cell proliferation and migration [87].

Previous studies demonstrated that curcumin as a hepatoprotective antioxidant inhibited the proliferation and metastasis of HCC [88]. Khan et al. combined curcumin with N-n-butyl haloperidol iodide (F2) to form an F2C combination and validated it in an *invitro* xenograft model. They found that the F2C combination inhibited the interaction between EZH2 and H19 in regulating Wnt/β-catenin signaling in HCC, thereby suppressing HCC development [89].

Overall, previous studies suggest that EZH2 is a valuable target for anti-HCC therapy. Future studies should further investigate therapeutic approaches to control HCC progression through inhibition of EZH2.

## Conclusion and discussion

As one of the core catalytic subunits of PRC2, EZH2 exerts its tumor-promoting function in HCC mainly by mediating histone methylation in the promoter region. In general, EZH2 promotes the proliferation of HCC cells, activates EMT pathway, inhibits the function of immune cells and participates in immune evasion in HCC cells.

In HCC-related tumor immunity, studies have revealed that EZH2 negatively regulates NK and T-cell antitumor responses. The role of EZH2 on B cells in HCC remains unclear, only in the study by Wei et al., the expression of EZH2 is involved in the pathway whereby B cells are converted to IgG-producing plasma cells in HCC tissues, inhibiting macrophage production of cytokines that reduce antitumor immune responses [78]. In addition, some studies reported that EZH2 stabilizes PD-L1 in HCC cells through the IL-6/JAK1 pathway [60], however, in the study undertaken by Liu et al. study, only 30% of tissues stained positive for PD-L1 expression. Subsequently, the authors demonstrated that EZH2 negatively regulates PD-L1 expression by inhibiting downstream factor IRF1 in hepatoma cells treated with IFN-γ *ex vivo* [90, 91]. These results seem contradictory and require further research.

As many mechanisms of EZH2 in the occurrence and development of HCC remain uncertain, further research is needed. The purpose of exploring the function and mechanism of EZH2 in HCC is to find effective therapeutic strategies against HCC. Studies have demonstrated that EZH2 also plays a role in 5-FU and sorafenib resistance in chemotherapy treatment for HCC. In addition, considering the role of EZH2 in the treatment of HCC, research has found that resistance to the pan-FGFR inhibitor Infigratinib often occurs in long-term treatment of HCC. This may be due to reactivation of EZH2, up-regulating the ErBb family and FGFR2-4 [23]. Combining an EZH2 inhibitor with Infigratinib may be conducive to development of drug resistance, however, there is currently no FDA-approved drug targeting EZH2 to treat HCC. Therefore, it is necessary to develop EZH2 inhibitors with low side effects and high efficiency: more research should be undertaken into the use of EZH2 inhibitors alone (or in combination) in the clinical treatment of tumors to verify the effects of different combinations for HCC.

## Data Availability

Not applicable.

## Abbreviations

HCC: hepatocellular carcinoma
PRC2: polycomb repressive complex 2
US: ultrasound
MRI: magnetic resonance imaging
TACE: transcatheter arterial chemoembolization
RFA: radiofrequency ablation
GATA4: globin transcription Factor 4
AR: androgen receptor
PLZF: promyelocytic leukemia zinc finger protein
RORα: retinoic acid-related orphan nuclear receptor alpha
CHD5: Chromodomain helicase DNA binding protein 5
CXCR4: CXC chemokine receptor 4
FAK: focal adhesion tyrosine kinase
CXCL10: CXC motif ligand 10
HDAC10: histone deacetylase 10
ncRNA: non-coding RNA
CDK: Cyclin-dependent kinase
EMT: Epithelial-mesenchymal transition
FOXA2: forkhead box A2
HBV: hepatitis B virus
CCRK: cell cycle-related kinase
KC: Kuppfer cell
MDSC: Myeloid-derived suppressor cell
ARG-1: arginase 1
FBP 1: fructose-1, 6-bisphosphatase 1
EVs: extracellular vesicles
DZNep: 3-deazaneplanocin A
SAH: S-adenosylhomocysteine
SAM: S-adenosyl-L-methionine
B-NHL: B-cell non-Hodgkin lymphoma
DLBL: diffuse large B cell lymphoma; DNMT: DNA methyltransferase
IGF1R: Insulin-like growth factor 1 receptor
DAC: 5-Aza-CdR
OGT: O-linked N-acetylglucosamine (GlcNAc) transferase
ESE: emodin succinyl ester
20(S)-GRh2: 20(S)-Ginsenoside Rh2
IRF1: interferon regulatory factor 1
